# Advances in Genetic Tools and Their Application in *Streptococcus thermophilus*

**DOI:** 10.3390/foods12163119

**Published:** 2023-08-19

**Authors:** Ruiting Zhao, Zouquan Chen, Jie Liang, Jiaxin Dou, Fangyu Guo, Zhenshang Xu, Ting Wang

**Affiliations:** 1 State Key Laboratory of Biobased Material and Green Papermaking, Qilu University of Technology, Shandong Academy of Science, Jinan 250353, China; zhaoruiting12@163.com (R.Z.); 18827174212@163.com (Z.C.); liangjie122610@163.com (J.L.); 17864099662@163.com (J.D.); gfangyu2022@163.com (F.G.); wangting@qlu.edu.cn (T.W.); 2 School of Bioengineering, Qilu University of Technology, Shandong Academy of Science, Jinan 250353, China

**Keywords:** *Streptococcus thermophilus*, genetic tools, plasmid, promoters, production characteristics

## Abstract

*Streptococcus thermophilus* is a traditional starter. Nowadays, key aspects of *S. thermophilus* physiology have been revealed concerning the phenotypic traits relevant for industrial applications, including sugar metabolism, protein hydrolysis, and the production of important metabolites that affect the sensory properties of fermented foods as well as the original cooperation with *Lactobacillus delbrueckii* subsp. *bulgaricus*. Moreover, significant advances have been made in the synthetic biology toolbox of *S. thermophilus* based on technological advances in the genome and its sequencing and synthesis. In this review, we discuss the recently developed toolbox for *S. thermophilus*, including gene expression toolsets (promoters, terminators, plasmids, etc.) and genome editing tools. It can be used for both functionalized foods and therapeutic molecules for consumers. The availability of new molecular tools, including the genome editing toolbox, has facilitated the engineering of physiological studies of *S. thermophilus* and the generation of strains with improved technical and functional characteristics.

## 1. Introduction

*Streptococcus thermophilus* is widely used for its acidification ability in the dairy industry for making different fermented foods. It is mainly used in combination with lactobacilli for the production of processed cheeses, mozzarella, or Swiss-type hard cooked cheeses and as a costarter with *Lactobacillus delbrueckii* subsp. *bulgaricus* in the manufacture of yogurt. *S. thermophilus* is also used alone to produce fermented milks, particularly with the aim of producing functional foods, because in addition to lactic acid, it also produces low levels of formate, acetoin, diacetyl, acetaldehyde, and acetate as additional end products. 

The methods of metabolomics, metabolic engineering and genetic, recombination are current tools to reveal important genetic, genomic, and phenotypic characteristics of strains of microorganisms in the production of probiotics and functional foods. This is relevant for the management of fermentation processes in order to increase functionality or the beneficial influence on the health of the host. Extracellular polysaccharide production, protein hydrolyse and flavor ingredient production, acidification capability, and other crucial *S. thermophilus* production features all have significant effects on the caliber of dairy products [1]. The strain’s ability to reduce acidity controls both the amount of time it takes to produce a dairy product and its quality. This is dependent on how well the strain can use sugar. The growth environment of strains is in a constant process of change, sometimes requiring fermentation in environments containing different sugars, so the sugar utilization of *S. thermophilus* is closely related to its fermentation capacity and the range of applicability of the strain. In addition, some *S. thermophilus* are able to secrete extracellular polysaccharides (EPS) during the growth process, which help to improve the viscosity and texture of dairy products. Dairy products are more widely accepted when they have flavoring added. In addition to the consumption of nitrogen sources, the proteolytic activity of strains affects the generation of flavor components. Several strains have production characteristics that differ substantially from one another as a result of long-term evolution and environmental adaptability.

To establish heterologous expression of *S. thermophilus*, a variety of effective genetic tools, such as plasmids, promoters, terminators, and selection markers have been used. In addition, the discovery of *S. thermophilus*’ inherent capabilities has significantly increased its appeal as a potential new host for the creation of heterologous proteins. In contrast to other *L. lactis*, it offers the possibility to genetically engineer the bacterium using plasmids or linear DNA fragments. The creation of genome editing technologies is seen as a crucial step in improving our understanding of intricate cellular processes and phenotypic features with industrial application. Other biotechnological applications, such as cell factories by design and focused functional design utilizing synthetic biology principles, have been made possible by the availability of the genome editing toolbox. The use of genome editing, in contrast to traditional strain-enhancement methods or adaptive evolutionary approaches, permits the introduction of traits that may be challenging to select for or combinations of several traits that are uncommon in natural strains.

## 2. Advantages of *S*. *thermophilus* as a Host

### 2.1. Clear Genetic Background and Physiological Characteristics

The application’s safety can be increased by a clear genetic background. Whole-genome sequencing has emerged as a crucial tool for examining the LAB’s genetic make-up. In order to guarantee the safety of the strain and lay the theoretical groundwork for its use in actual production as a new food starter, this study assesses the safety of *S. thermophilus* from the perspectives of virulence gene prediction and analysis, antibiotic resistance analysis, hemolytic analysis, and transposition factor analysis and prediction. Prajapati et al. [2] discovered that the whole genome of *S. thermophilus* MTCC5461 contained inactivated or missing virulence-related genes. In contrast, the strain’s genome contained certain protein-coding genes that were preserved in the MTCC5461 and CNRZ1066 strains and associated with probiotic qualities (such as adhesion, acid resistance, bacteriocin synthesis, lactose utilization, etc.). Using whole genome sequencing technology, Alexandrak et al. [3] examined the *S. thermophilus* ACA-DC2 strain’s genome and discovered that it contained coding genes involved in the catabolism of lactose and galactose as well as proteolysis, which may be important for the strain’s adaptation to the environment of fermented milk. The majority of amino acid and peptide transporters, aminopeptidases, and amino acid biosynthetic pathways were discovered to be conserved in all strains, indicating their central role for the species, according to Alexandraki’s analysis of 23 fully sequenced genomes of *S. thermophilus* [4]. This will help to clarify significant aspects of strain diversity within this starter culture, which may be related to its use in the dairy industry. 

A key method for examining the evolutionary traits of the genes in *S. thermophilus* is comparative genomic analysis, a novel approach to investigate critical functional gene mining, microbial genome diversity, and horizontal gene transfer among microorganisms. Based on comparative genomic analysis, Bolotin [5] and other researchers have demonstrated that *S. thermophilus* strains typically require a milk environment to grow. To adapt to this growth condition, a number of harmful genes in its genome have gradually been degraded into pseudogenes or lost, resulting in a probiotic strain that is not harmful to humans or the environment. The multi-locus sequence typing (MLST) method can be used to clarify the phylogenetic relationships and evolutionary history of isolates. Finding a link between strains and their origin was an intriguing use of the MLST technology. The evolutionary relationships of 27 strains of *S. thermophilus* with *S. salivarius* and *S. vestibularis* were examined using the MLST technique by Delorme et al. in 2010 [6]. An improved knowledge of *S. thermophilus* genome evolution can be achieved through analysis of a geographically diversified and representative collection of isolates using MLST, as well as information for further research on the structure and genetic evolution of *S. thermophilus* globally [7].

The ability of strains to adapt to their environment, survive in hostile environments, and carry out their regular functions can all be fundamentally improved by using transcriptome analysis to explain the regulation rules of stress tolerance mechanisms in the face of hostile environmental stresses. A theoretical framework for gene-level transformation of *S. thermophilus* can be established by elucidating the genetic background of a newly isolated strain of *S. thermophilus* with excellent and distinctive characteristics, as well as the laws governing gene-level regulation of various traits related to production efficiency and product quality. This information allows a more in-depth study of the genetic structure of many metabolic activities of the species, such as amino acid metabolism [8], alignment of proteolytic systems [8], phage resistance [9], folate biosynthesis [1], urea metabolism [10], and biofilm formation [11]. The quality of fermented dairy products is determined by the production features of *S. thermophilus*, including acidification ability, proteolytic activity, formation of EPS, bacteriocins and flavor compounds, phage and host defense capacities, etc. The genomes of various strains with various production traits typically differ significantly from one another. Significant changes were the manufacture of bacteriocin and EPS, peptide metabolism, genes involved in phage resistance, etc. The development of research on *S. thermophilus* primary production traits and molecular level would boost knowledge of the molecular mechanisms underlying strains with various crucial traits and raise the bar for industrial control of fermented foods.

1. Sugar metabolism

**Figure 1:**
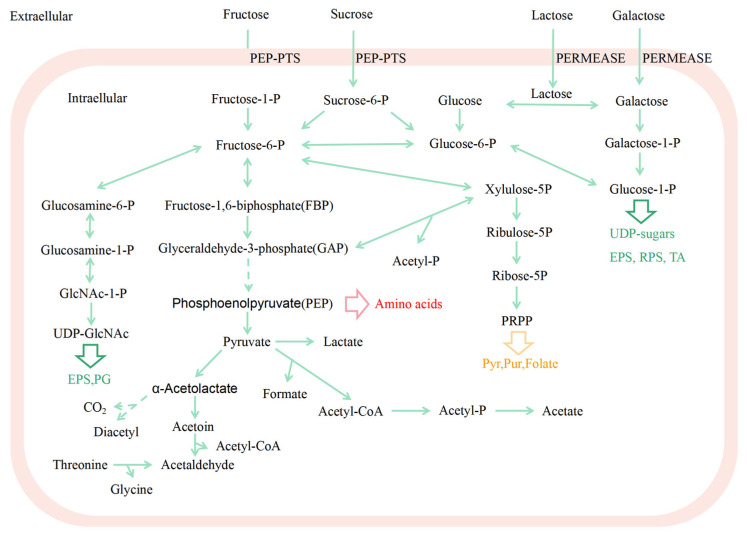
Pathways of sugar metabolism in *S*.*thermophilus*. Fructose-1P, fructose-1-phosphate; Fructose-6P, fructose-6-phosphate; FBP, fructose-1,6-biphosphate; Suc-6P, sucrose-6-phosphate; Glucose-6P, glucose-6-phosphate; Glucose-1P, glucose-1-phosphate; GAP, glyceraldehyde-3-phosphate; PEP, phosphoenolpyruvate; Acetyl-P, acetyl-phosphate; Xylulose-5P, xylulose-5-phosphate; Ribulose-5P, ribulose-5-phosphate; Ribose-5P, ribose-5-phosphate; PRPP, 5-phosphoribosyl diphosphate; UDP-GlcNAc, UDP-N-acetyl-glucosamine; Pyr, pyrimidines; Pur, purines; EPS, exopolysaccharides; PG, peptidoglycan; RPS, rhamnose polysaccharides; TA, teichoic acids; PTS, sugar phosphotransferase system. Arrow indicates a reaction, multiple enzymatic processes are shown by dashed arrows, double-headed arrow indicates a bidirectional reaction. The figure is original.

The milk acidification rate of *S. thermophilus* is significant technically in the commercial fermentation process. The strain’s capacity to utilize sugar determines how quickly it becomes acidic. Numerous investigations have demonstrated that the majority of *S. thermophilus* strains are capable of using lactose. Because milk contains a particular enzyme called lactose permeating enzyme, which is produced by the *lacS* gene, lactose is the primary carbohydrate present in milk. Once lactose enters the cell, galactosidase (encoded by lacZ) will effectively hydrolyze it to produce glucose and galactose. Glucose is typically directly digested by glycolysis in *S. thermophilus;* however, galactose is not metabolized. The genetic basis for *S. thermophilus*’ inability to metabolize galactose has also been identified. However, van den Bogaard et al. [12] discovered that galactose metabolism (Leloir pathway) (gal K-TEM) is also present in galactose-metabolism-negative strains of *S. thermophilus*, despite the majority of these strains being unable to utilize galactose. It was discovered that, when compared to the genes of positive strains, the majority of *S.* thermophilus strains’ poor expression of galactokinase was the key factor restricting their ability to metabolize galactose. Sucrose and fructose are the only sugars that are transported by photosphosphoenolpyruvate-dependent photosynthetic systems (PTS) [13]. *Streptococcus thermophilus* does not like the non-PTS sugar glucose as a substrate. Glucokinase phosphorylates it to produce glucose-6-phosphate, which is then further processed by the enzyme glycolysis (Figure 1). The glucokinase missense mutation rendered the mutant incapable of fermenting glucose. Galactose, mannose, sucrose, maltose, meliose, and raffinose, on the other hand, are only used by a smaller number of strains [14,15]. More and more food matrices, such as natural yogurt, sweetened yogurt, and vegetable products, are including *S. thermophilus* strains. They come across more types of sugars than lactose in these items, typically in the form of combinations.

2. Polysaccharide biosynthesis

Most strains of *S. thermophilus* have the ability to create EPS. Also known to produce CPS are some strains of *S. thermophilus* [16]. In the course of bacterial development, the former produces EPS in the cell through a difficult series of interactions between *S. thermophilus* intracellular enzymes, and then exports it to the extracellular environment as a macromolecule through a unique lipid carrier, where the latter transiently binds to the cell surface. The EPS biosynthesis pathway includes the transport of glucose to the cytoplasm, the creation of glucose-1-phosphate, polysaccharide production, and the generation of EPS [17]. The two types of EPS are homoglycans and isoglycans; the former are composed of just one kind of sugar, whereas the latter are made up of a variety of sugars. Galactose, glucose, and rhamnose make up the majority of isosaccharides, but N-polymers containing acetylated galactosamine, fucose, and galactose have also been discovered.

The relevant genes for the intricate biochemical process of bacterial EPS production are found in the eps gene cluster of bacterial chromosomes or plasmids. On chromosomes, *S. thermophilus* has all of its EPS gene clusters. EPS-producing genes (*epsA*, *epsB*) and EPS chain-length genes (*epsC*, *epsD*) are regulated in the first region of the *S. thermophilus* EPS cluster, which is divided into three parts. The second region is involved in the biosynthesis of EPS repeating units, and the third area is in charge of repeating unit aggregation and exit [18,19]. At least 20 different forms of EPS gene clusters have been discovered, and *S. thermophilus* EPS clusters have been shown to be highly diverse and range in size from 15–36 kb. The genetic diversity of EPS clusters has a substantial impact on the structural alterations of EPS molecules. In general, the relationship between an EPS’s structure and function is strong. Therefore, EPS’s highly polymorphic structure provides a wide range of potential applications. *Streptococcus thermophilus* EPS can be utilized as a thickener, stabilizer, emulsifier, gel, and water binder in food due to its GRAS (generally recognized as safe) and probiotic qualities.

3. Protein metabolism

**Figure 2:**
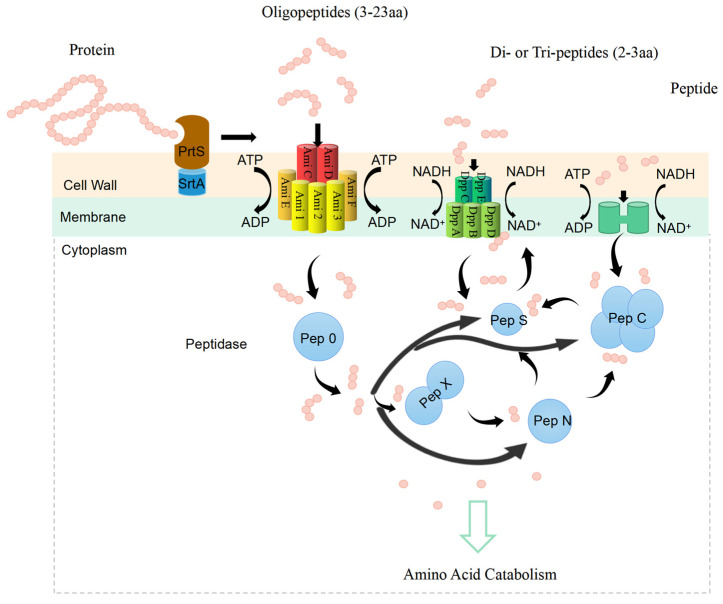
*S. thermophilus* proteolytic system. There are three steps in the system. Proteins are broken down in the first stage by a proteinase called PrtS, which Sortase A (SrtA) anchors to the cell wall. The second step is transport of peptides and oligopeptides. The transport of peptides is integrated by the Dpp system with five proteins (DppA, DppB, DppC, DppD, and DppE) regulated by ATP and proton formation (NAD+); the oligopeptides transport is carried out by the Ami system integrated in an operon system with seven proteins (Ami1, Ami2, Ami3, AmiC, AmiD, AmiE, and AmiF) regulated by ATP and activated by sulfur amino acid presence. The peptide ComS controls the transcriptional regulatory protein ComR linked to the Ami system. The third and final stage is the cutting of peptides by fourteen internal peptidases, five of which (PepO, PepS, PepX, PepN, and PepC) differ from those of other LAB in terms of their properties and specificity. The figure is original.

According to Figure 2, the proteolytic system of *S. thermophilus* can be broken down into three main sections. The first section involves the degradation of proteins by the serine protease PrtS, which is typically stabilized in the cell wall by the particular binding protein SrtA. Peptides and oligopeptides are then transported, typically by the Dpp and Ami systems. The third component is the metabolism of peptides by intracellular peptidases, among which *S. thermophilus* has unique needs for the peptidases PepO, PepS, PepX, PepN, and PepC. Galla et al. [20] evaluated the proteolysis and acidification characteristics of 30 *S. thermophilus* strains isolated from yoghurt or cheese. Out of the 30 strains examined, 12 displayed cell envelope-associated protease activity (PrtS), 3 displayed a slight PrtS activity (PrtS^++/−^), and 15 displayed no PrtS activity (PrtS^−^), even though 8 of them possessed the appropriate gene (*prtS*). The decrease of PrtS activity in the PrtS strains may be caused by altered *prtS* regulation, according to the sequencing of the *prtS*gene in four PrtS^−^ and one PrtS strains. According to earlier research, the strain’s high proteolytic activity and high acidizing capacity are closely related. These findings suggest that the strain grows more quickly and produces acid in milk when there is strong protease activity.

The phenotypic traits of the several 16S-23S intergenic spacer (ITS) region sequence types are the same. ITS-St-I, ITS-St-II, ITS-St-III, and ITS-St-IV are among the 90 varieties of *S. thermophilus* that have so far been identified [21]. Common types are ITS-St-I and ITS-St-II. According to Galla et al. [20], only six strains possessed ITS-St-V alleles, whereas the majority of strains had ITS-St-I (10 out of 30) or ITS-St-II (6 out of 30) alleles. In general, strains with the ITS-St-III allele displayed greater protease-digesting and acidification capacities, whereas strains with the ITS-St-I allele displayed little to nil PrtS activity.

### 2.2. Food-Grade Safety

The food sector makes extensive use of *S. thermophilus*. Numerous studies have demonstrated its safety, healthfulness, and lack of production of harmful chemicals. Tools for systematic gene editing, which is necessary for molecular change to reach the food level and is non-toxic and safe for the human body, must be available. *S. thermophilus* is frequently employed in the making of cheese and yoghurt and may have been since humans first engaged in the dairy business, demonstrating the species’ safety as a food microbe. Additionally, a genome analysis revealed that *S. thermophilus* lacked, or had inactivated or missing, virulence-related genes. 

Pathogenic streptococcus must be able to utilize significant amounts of carbohydrates in order to be virulent, probably because it allows the bacteria to maintain their ecological niche. *S. thermophilus* has been reported to have a deficiency in this function, which may reduce its pathogenic potential. Furthermore, it appears that *S. thermophilus* has lost the bulk of the streptococcal virulence-related genes, including those involved in cell adhesion, host invasion, or immune system evasion, as a result of the evolution of loss-of-function events that represent the dairy niche. Antibiotic resistance has a significant impact on pathogen pathogenicity as well.

Many streptococcal virulence-associated genes (VRGs) are absent from the *S. thermophilus* genome or present solely as pseudogenes unless they produce proteins that conduct crucial cellular functions. The *S. thermophilus* genome does not appear to include any antibiotic-modified genes. The *S. thermophilus* genome was changed by evolution by choosing the strains that grew the best in milk, as demonstrated by in-depth computer analyses of its cell metabolism. *Streptococcus pneumoniae* and *Streptococcus pyogenes* both include more than a quarter of the virulence-associated genes lacking in *S. thermophilus* (25/92), with over 40% (9/25) of these present in places where the two genomes are coterminous. This shows that virulence-associated genes were present in the ancestral strains of *S. thermophilus* as well as the pathogenic species, but they were lost from the latter.

The synthetic biology chassis is a recipient microbial cell that can be genetically modified and introduced into components. It can provide various features for cell growth and component work, and is stable enough for application in various fields. For intracellular heterologous expression, Gram-negative bacteria and *Escherichia coli* are most frequently utilized as chassis, while Gram-positive bacteria and *Bacillus subtilis* are preferred for heterologous secretion [22,23]. These bacteria are very adaptable to laboratory conditions and have great performance, large design space, genetic sites, and regulatory elements. They also have rapid reproduction and stable protein synthesis. Antibiotic resistance genes are primarily used as selection markers in genetic engineering operations, but in one-step plasmid-free transformation, it is possible to introduce foreign DNA into cells and integrate it into chromosomes through homologous recombination, which runs the risk of being transferred to environmental microorganisms. *Bacillus subtilis* [24] was expressed using LAB as an alternate host. Numerous gene modification tools, effective protein secretion, and a small number of expected cell surface proteins are only a few benefits of *L. lactis* [25]. It also possesses only one surface housekeeping protease, HtrA, which is capable of destroying abnormally produced proteins. Finally, unlike *B. subtilis*, the species can be utilized to research protein behavior in food (in situ) or in vivo because it is commonly found in dairy products and has a recognized safety (GRAS) status. However, compared with other LAB species such as *S. thermophilus* [26], *L. lactis* still lacks natural capabilities. Through the use of the species’ DNA caption route and this capacity to internalize foreign DNA, it is possible to alter the species and introduce foreign genes through plasmid builders or directly into chromosomes. In actuality, this technique permits both the use of linear pieces and the transformation of *S. thermophilus* with conventional plasmid vectors. Assume that the upstream and downstream sections of the foreign DNA require the insertion of two 1000-base (kb) homologous DNA pieces, one on each side. They can then be integrated directly into the chromosome [27] if that is the case. Additionally, the utilization of linear pieces permits the insertion of large DNA fragments up to 15 kb, which is a significant issue for plasmid vectors [28]. As a result, *S. thermophilus* has an advantage over LAB species that are still incapable of spontaneous transformation thanks to this ability.

## 3. DNA Components

### 3.1. Promoters

The promoter controls the amount of gene transcription and is a critical cis-acting region for gene expression. The level of gene expression is directly impacted by the promoter’s activity. In the transformation process of the microbial metabolic engineering pathway, a common strategy is to use promoters with different strengths to finely regulate the expression of target genes, so as to achieve the coordination and balance between the generation and utilization of intermediate metabolites, and improve the synthesis efficiency of metabolites. In a specific synthetic pathway, different genes need to be expressed in different degrees to achieve the balance of each metabolic branch and ultimately improve the yield of the target product. However, most natural promoters have difficulty meeting the requirements in terms of both strength and flexibility. Based on the limitation of natural promoters in application, transforming and screening new synthetic promoters is particularly necessary. In 2005, Alper et al. [29] first mentioned the concept of promoter engineering, pointing out that promoters can be modified in ways such as protein engineering to create promoter libraries with a wide range of genetic regulation, leading to systemic improvements. The main research content in the field of promoter engineering is to overcome the defects in the application of natural promoters, transform and screen promoters with good functions that meet the needs of metabolic engineering research, regulate gene expression more flexibly and finely, and provide a powerful regulatory tool for the pathway transformation of metabolic engineering.

Different promoters, often constitutive and inducible promoters, are accessible in *S. thermophilus* for metabolic engineering and synthetic biology. Without induction or inhibition, constitutive promoters offer continuous expression levels. In contrast, inducible promoters give the user significant control over the timing of gene expression. When the gene loads the cell at the time of expression, this control is desirable. Before selecting to utilize an inducible promoter, however, a number of parameters should be taken into account, including the cost of metabolite-induced expression, the promoter’s sensitivity to metabolites, the timing of induction, and the background expression in the absence of metabolites.

Promoters that have been removed from the genome are the most frequently used (Table 1). Some of these, such as promoter STP2201 [30] and promoter P25 [31], have been created to be active in *S. thermophilus* and *E. coli*. Some promoters, like the one for the *hlbA* gene in *L. delbrueckii subsp. bulgaricus* ATCC 11,842 [32], permit robust and constitutive expression in *S. thermophilus*. Recently, the cell envelope protease PrtH from *L. helveticus* was heterologously expressed via the promoter of the *prtS* gene, which codes for the cell envelope protease of *S. thermophilus*. According to expectations, the heterologous gene’s transcript levels were almost identical to those of the *prtS* gene in the wild-type strain [33]. Since these promoters were created using reporter red fluorescent proteins for identification in *S. thermophilus*, *Lacticaseibacillus casei*, and *E. coli* [34], it is known that the first library of constitutive promoters for *S. thermophilus* is currently available.

### 3.2. Terminator

The terminator region, which is essential for mRNA half-life and ending transcription, is another essential element of genetic control. However, practically all applications of synthetic biology and metabolic engineering use a small number of common terminators. In contrast to promoters, terminators have not been thoroughly researched despite their relevance; for instance, few studies have looked at how terminators affect net protein expression. However, terminators have been demonstrated to influence the stability and abundance of their respective mRNAs [41], and these findings suggest that these components should be similarly taken into account as part of the synthetic biology toolbox.

The most extensive collection of intrinsic terminators from completely sequenced bacterial genomes and plasmids is included in WebGeSTer (Table 2). An enhanced version of GeSTer called WebGeSTer was used to create the database. The 1060 bacterial chromosomes and 798 plasmids found in the NCBI database that have intrinsic terminators are all included in the WebGeSTer DB. The database contains data on a total of 977 173 terminators, both canonical and non-canonical [42]. *S. thermophilus* is shown in the following Table 2.

(i) L-shaped (canonical terminators): a trail of 10 bp that contains more than three uridylates follows a hairpin.

The following four types of non-canonical terminators:

(ii) I-shaped: where the path after the hairpin contains three uridines;
(iii) U-shaped: multiple hairpin structures working together, with around 50 nt between them;
(iv) X-shaped: convergent structures of the right and left strands that serve as terminators for the convergently transcribed genes; and
(v) V-shaped: two hairpins, the second of which begins right after the first.

## 4. Plasmid Vectors

### 4.1. Non-Food Grade Expression Vectors

Genes with resistance-selecting markers are typically ligated to vectors to produce the appropriate specific strains when specific strains are desired. The common resistance screening genes in these vectors, erythromycin and chloramphenicol, are easily transferred to the environment and endogenous microorganisms, creating a significant biosafety issue. Such vectors are typically non-food-grade expression vectors. From the perspective of safety, in order to avoid the transfer of antibiotic resistance genes to the environment or other organisms, the U.S. FDA and other authorities have banned the use of antibiotic-based selection markers for GRAS microorganisms. A selective marker system that fails to meet this standard will restrict lactic acid LAB’s more profound development and utilization.

### 4.2. Food-Grade Expression Vectors

To prevent issues with food safety during production, for consumption by humans the vector must be a food-grade LAB expression vector with distinct genetic properties and stable inheritance. *Streptococcus thermophilus* food-grade vectors have been studied for use in food production in recent years, and food-grade selection marker genes have been inserted into the vectors. For *S. thermophilus*, various non-antibiotic resistance selection markers have recently been created with success. According to the phenotypic relationship and characteristics between plasmid and recipient bacteria at the time of screening, they are mainly divided into dominant and complementary selection markers.

#### 4.2.1. Dominant Selection Marker

Dominant selection markers are used to select transformants by transferring plasmids with selection marker genes to give new characteristics to the recipient bacteria so they can grow and reproduce under the corresponding selection pressure. These selection markers are easy to use and do not require prior genetic modification of the recipient bacterium. Studies on non-antibiotic resistance markers in *S. thermophilus* molecular technology have mainly involved heavy metal resistance and heat-activated protein-dominant selection markers.

Wong et al. [43] constructed a pND919 non-antibiotic resistant plasmid vector using *S. thermophilus* replicon and *cadA* and *cadC* genes of *L. lactis* and transferred it into *S. thermophilus* ST3-1 and ST4-1. The growth of this sensitive non-resistant strain was inhibited in a medium containing 0.3–0.5 mmol/L CdCl_2_ during culture, and the resistant transformants were screened. Under selection pressure, the plasmid pND919 remained stable after 40 generations of transmission. This plasmid was also the first safe vector applied to the engineering strain of *S. thermophilus*. The marker gene with cadmium resistance was used in both *L. lactis* and *S. thermophilus*.

The *shsp* gene in *S. thermophilus* encodes a heat-stimulated protein (Table 3). After the plasmid containing *shsp* was transferred, the resistance of the strain to heat and acid was significantly enhanced, and the optimum growth temperature was also reduced [44], so that the strain could survive under relatively extreme conditions. The plasmid pSt04 with *shsp* was isolated from *S. thermophilus* S4 strain, which contained the open reading frame of *shsp*, and the expressed protein had high homology (>90%) with the small heat shock protein in other strains. The non-antibiotic-resistant plasmid pSt08 with *shsp* and restriction–modification system (R/M system) was further constructed and transferred into *S. thermophilus* strains. The transformants could be grown at 60 °C or 52 °C at pH 3.5 for screening. For *S. thermophilus* strains and other *L. lactis* strains, *shsp* is a promising, safe non-antibiotic resistance selection marker.

#### 4.2.2. Complementary Selection Markers

The recipient strains of complementary non-antibiotic resistance plasmid selection markers usually have mutations in genes related to metabolic pathways, resulting in mutant strains that cannot grow on basic media. The strain can only survive if supplemented with the appropriate exogenous nutrients or transferred to a plasmid containing a specific selection marker gene. 

Sasaki et al. [45] constructed food-grade vectors by using *thyA* as a selection marker for *S. thermophilus* mutants requiring thymidine. Thymidylate synthase is a key enzyme in the thymidylate synthesis pathway and is encoded by *thyA*. The gene *thyA* derived from the *L. acidophilus* strain can be used as a selection marker to construct plasmids. It was found that *S. thermophilus* strain TM1-1 was obtained by subculturing the strain in a medium containing methamphetamine, and two *thyA* genes, *thyA_St_* and *thyA_Lb_*, isolated from *S. thermophilus* and *L. delbrueckii* subsp. *bulgaricus*, were used to construct plasmids for *S. thermophilus*. The constructed plasmid was transferred into the host bacteria, the screening was as effective as the erythromycin resistance screening, and the exogenous amylase gene (*amyA*) was successfully expressed using this plasmid. The results showed that *thyA* gene is a safe and effective non-antibiotic resistance selection marker and can be used to express exogenous protein safely.

The α-galactosidase selection marker was used to replace the chloramphenicol resistance gene initially contained in the pRAF301 plasmid by using the α-galactosidase gene (*aga*) isolated from *L. raffinolactis* ATCC 43,920 strain [46]. It was also transferred into *S. thermophilus*, and after incubation at 37 °C in medium with melibiose and raffinose as the main carbon source, the transformants were screened as effectively as chloramphenicol resistance markers.

### 4.3. Endogenous Plasmid Vectors

As self-replicating DNA molecules, although plasmids lack the genetic material required for bacterial survival, they frequently carry particular genes that provide the strain with beneficial properties, such as the ability to use casein, synthesize EPS, transport potassium, be phage-resistant, and produce bacteriocin. *S. thermophilus* strains have fewer endogenous (Table 4) plasmids than some LAB species, like *Lactiplantibacillus plantarum* and *L. lactis* [56], which have several plasmids. The majority of *S. thermophilus* plasmids lack clear phenotypic features, making them cryptic in nature. Some plasmids have been found to encode small heat shock proteins, including pER341 [57], pCI65st [58], pND103 [59], pST04 and pER1-1 [60], pt38 [61], pER7, pER16, pER26, pER35, pER36, pER41 [53,62], pK1002C2, and pK2007C6 [63]. The development of these tiny heat shock proteins improves the heat and acid tolerance of strains containing heat shock proteins, according to numerous studies that have demonstrated how they are activated by high temperature and low pH. As a result, the possible application of the heat shock protein gene promoter hsp16.4 of pER341 in the temperature-controlled production of heterologous genes in LAB is being explored. Genes relevant to the restriction modification system can be found in the plasmids pCI65st [58], pSt08, pSt0, and pER35 [62]. 

The majority of *S. thermophilus* plasmids replicate via rolling circle replication (RCR). The host range, stability, and copy number of the plasmid source vector are only a few of the crucial properties that are intimately related to the plasmid replication mode. RCR plasmids often have several copies, are structured neatly, and are tiny (less than 10 kb). Plasmids, as opposed to RCR plasmids, have more structural stability. The majority of *S. thermophilus* RCR plasmids are members of the pC194 family; however, pSMQ172 is a member of the pE194/pMV158 family. Despite being smaller than 10 kb, the plasmids pSMQ-316 and pSMQ-312b reproduce using the θ replication mode [64]. *Streptococcus thermophilus* can use a wide variety of expression vectors, including free or integrated vectors that antibiotic resistance genes or food-grade indicators can select thanks to the development of these technologies. Additionally, they can be repeated in *E. coli* or other LAB species if necessary.

## 5. Genome Editing Tools

**Figure 3:**
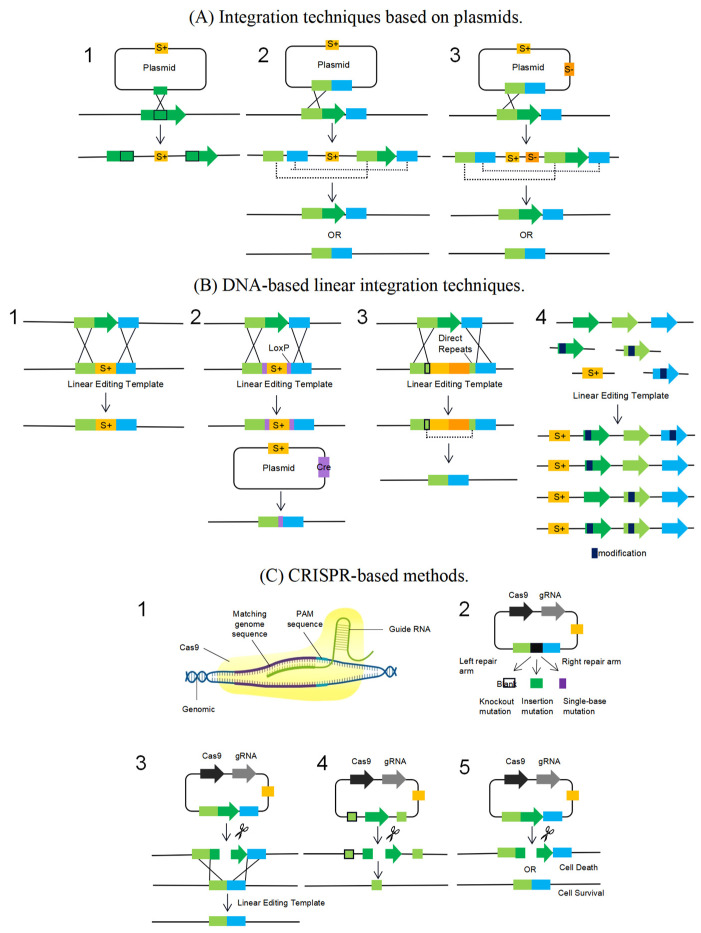
Overview of genome engineering tools for *S. thermophilus*. (**A**) Integration techniques based on plasmids. (**B**) DNA-based linear integration techniques. (**C**) Process flow diagram for gene editing using the CRISPR/Cas9 system, CRISPR-based methods. The description of the details in this figure has been added as follows. Arrows represent target genes. The green rectangle (_▀_) represents left repair arm. The blue rectangle (_▀_) represents right repair arm. S^+^, positive antibiotic selection marker. S^−^, negative selection marker. The black rectangle (^█^) represents modification. gRNA-Cas9, active complex of a guide RNA and the RNA-guided endonuclease Cas9. The figure is original.

Other biotechnological applications, such as cell factories by design and focused functional design utilizing synthetic biology principles, have been made possible by the availability of genome editing toolboxes. Comparatively speaking, *S. thermophilus* lacks advanced and accurate genome editing techniques. The development of such tools has been hampered from an industrial standpoint by regulatory limitations on the use of recombinant DNA technology to alter microbes for food purposes. The most popular technique for eradicating genomic deletions in *S. thermophilus* is plasmid-based homologous recombination (Figure 3(A1)). Although these tools are effective, there are some defects. The requirement for antibiotic selection markers restricts the number of alterations per cell and may have an impact on the mutant’s physiology. An approach using only a single cross event may result in polarity effects altering downstream gene expression (Figure 3(A2)). These restrictions can be circumvented by dual-exchange events (Figure 3(A3)) [10,71], but they take longer because the second exchange event can bring back the wild-type phenotype. Reverse selection markers, such as tRNA phenylalanine synthetase [72] encoding orotic acid transporter [73,74] or mutant forms of *oroP* can help with the recovery of double-exchange events. These negative selection markers can be adapted to a range of bacteria, including *S. thermophilus*, because they are not dependent on particular background genotypes.

It has been established that *S. thermophilus* exhibits natural competence, a momentary cellular activity characterized by the uptake of exogenous DNA through membrane transport proteins [75]. This mechanism is combined with homologous recombination to achieve precise deletion of genes by replacing target genes with linearly edited templates expressing positive selection markers (Figure 3(B1)). The potential of this method for extensive genome alteration has been demonstrated by the use of natural ability to introduce a complete manipulator expressing the cell envelope protease PrtS (15 kb) in *S. thermophilus* strains without PrtS (Figure 3(B2)). By including loxP sites on either side of the positive selection marker in the linear editing template, Fontaine et al. [76] considerably increased the efficiency of gene deletion. The selection marker is excised by plasmid-expressed Cre recombinase after genome integration, leaving no trace of the marker. By incorporating a negative selection marker and two flanking direct repeat sequences in the linear editing template, a similar technique is described in *S. mutans* [77] to get rid of the recombinase necessary for marker excision (Figure 3(B3)). By merging numerous unlinked linearly modified templates during cotransformation, as in *Vibrio* and *S. pneumoniae* [78], the natural ability can be leveraged for genome editing. A sophisticated library of mutants is produced as a result of this method, which can be utilized to hasten the optimization of metabolic pathways or phenotypes (Figure 3(B4)).

*Streptococcus thermophilus* has a large number of CRISPR systems, one of which, type II Cas9 nucleic acid endonuclease, has been successfully used for eukaryotic genome editing. It also goes by the name “gRNA-Cas9 complex system” due to the fact that the majority of how it works relies on gRNA-Cas9 compounds, which not only selectively detect DNA fragments and PAM but also cause DNA double-strand cleavage at the target region (Figure 3(C1,C2)). *Streptococcus thermophilus* CRISPR/Cas loci exhibit a wide range of variation during a protracted phase of vaccination and evolution. Based on changes in genomic location, repeat sequences, and *cas* genes, all of which are present in three different types of CRISPR-Cas systems, they are divided into four categories: CRISPR1, CRISPR2, CRISPR3, and CRISPR4. Additionally, due to variations in available space, CRISPR sequences fluctuate from strain to strain. Smaller Cas9 fragments, greater specificity, and a decreased off-target efficiency are all characteristics of the *S. thermophilus* CRISPR-Cas9 system (StCas9). The major step involves creating a functional vector using the host cell’s StCas9 system sequence and performing expression experiments. The design of the sgRNA, which consists of two complementary single-stranded oligonucleotides made of crRNA and tracrRNA, is the most crucial phase. Additionally, due to variations in available space, CRISPR sequences fluctuate from strain to strain. Smaller Cas9 fragments, greater specificity, and a decreased off-target efficiency are all characteristics of the *S. thermophilus* CRISPR-Cas9 system (StCas9) [79]. 

The literature reports that, by introducing a self-targeting spacer sequence into the plasmid vector [80], the endogenous CRISPR system can be used to eliminate mobile genetic elements. A vector that expresses *S. pyogenes* Cas9 and specific spacer sequences targeted mobile genetic factors in *L. lactis*. Substantial genome editing of *Streptococcus* can be approached similarly because of the prevalence of mobile genetic elements and their important contribution to phenotypic features that are relevant to industry (Figure 3(C4)). Recently, linear editing templates were integrated into CRISPR-based self-targeting in *S. mutans* by natural transformation [81]. Combining CRISPR-based self-targeting with integration through a naturally altered linear editing template increases integration efficiency (Figure 3(C3)). Due to bacteria’s low capacity to repair double-stranded DNA breaks, the CRISPR system also serves as a counter-selection weapon. The method depends on the simultaneous transformation of plasmids expressing self-targeted CRISPR arrays and an editing template that includes the DNA cleavage site. This combination increases integration efficiency, and the CRISPR system also functions as a counter-selection weapon because bacteria have a limited capacity to repair double-stranded DNA breaks (Figure 3(C5)). Applying this strategy to *S. thermophilus* appears conceivable given the successful development of numerous endogenous CRISPs. Combining genome editing techniques with molecular tools for heterologous protein expression may improve our comprehension of intricate cellular processes and open the door to brand-new industrial uses for *S. thermophilus*. The development of *S. thermophilus* strains with superior technical and/or functional properties is made possible by the accessibility of novel molecular tools, including as toolkits for genome editing.

## 6. Application of Engineered Strains of *S. thermophilus*

Due to the accelerated pace of life, diseases such as gastrointestinal inflammation, obesity, and diabetes have become prevalent in today’s society. Common treatments such as medication and surgery are costly, risky, and have certain side effects. The development of synthetic biology of *S. thermophilus* offers the possibility of disease treatment and drug delivery. Currently, specific genes against diabetes, gastrointestinal diseases, and other illnesses can be inserted into the chassis, and drug properties can be embodied in dairy products using the fermentation principle, which is practical and effective while enhancing the taste, providing new ideas for food and drug development (Table 5). The streptococcal cloning vector pIL253 was used to introduce the *Streptomyces* antibioticus tyrosinase (*mel*) gene into *S. thermophilus* [82]. Vaillancourt et al. [83] demonstrated that *S. thermophilus* SMQ-301 was able to grow on galactose when complemented with the *S. salivarius galK* gene cloned on a low-copy plasmid. Excess galactose in dairy products may also adversely affect human health, particularly in individuals with galactosemia. The accumulation of galactose in dairy products is reduced to mitigate adverse effects. Chaves et al. [84] constructed a strain of *S. thermophilus* and cloned the *glyA* gene under the control of a strong promoter (P (LacA)). It can be used to control and improve the production of acetaldehyde in fermentation (dairy products) using *S. thermophilus* as the starting culture. The 11-residue antimicrobial peptide from bovine lactoferrin (BL-11) and the 12-residue hypotensive peptide from alpha(s1)-casein (C-12) have now been cloned in *S. thermophilus* to develop strains that enhance the functionality and nutritional value of dairy food products [85]. Del Carmen et al. [86] enhanced the anti-inflammatory activity of an immunomodulatory strain of *S. thermophilus* by conferring on it the capacity to produce antioxidant enzymes. The research results suggest that LAB strains that are able to modulate immune responses and that also express antioxidant enzymes could be useful in the development of novel therapeutic products that prevent IBD.

Multiple examples of bioactive EPS and its derivatives from *S. thermophilus* have been described for their antibacterial and antioxidant activity [39]. It has been demonstrated that many *S. thermophilus* strains produce EPS. Sulfonation [87], oxidation [88], and selenylation [89] of polysaccharides can result in the creation of novel pharmacological compounds with therapeutic uses [90]. Because it effectively increases the biological activity of polysaccharides, sulfonation is commonly utilized. Sulfated polysaccharides can exhibit higher solubility and may also have a number of enhanced biological activities, including antibacterial [91], antioxidant [92], antiviral [93], and anticancer activity [77], as well as anticoagulant qualities, by adding sulfated groups at the hydroxyl position.

## 7. Summary and Outlook

*Streptococcus thermophilus* is the only streptococcal species widely used in food fermentations, especially for yogurt manufacturing. Researchers now have the ability to characterize important technological and functional traits in a variety of *S. thermophilus* strains at the systems level thanks to the consolidation of genomics, the decline in the cost of genome sequencing, and the subsequent omics technology platforms (e.g., transcriptomics, proteomics, and metabolomics) that have emerged in recent decades. The selection and breeding of superior strains will be aided by knowledge of the probable molecular pathways underlying key traits of various strains.

As a result of extensive environmental adaptability and long-term evolution, different strains exhibit distinctly different production characteristics. A key long-term objective for academics and the fermenting business is to acquire new strains with distinctive and desired properties. Numerous research initiatives are being made to understand important facets of *S. thermophilus* physiology as a result of an increasing need for innovative starter cultures with increased functioning. Several phenotypic characteristics have connections to industrial uses. These processes include sugar metabolism, proteolysis, and the creation of vital metabolites that influence the organoleptic qualities of fermented foods and protocooperation with *L. delbrueckii* subsp. *bulgaricus*, including acetaldehyde, exopolysaccharides, and vitamins. The understanding of the molecular mechanisms of many strains with various significant characteristics will expand with advances in research on key production characteristics and molecular levels of *S. thermophilus*, which will also improve industrialization control for fermented foods.

Currently, there are still some issues with the application of *S. thermophilus* synthetic biology, and further study is still required to optimize the expression elements used in expression vector production. The emergence of new molecular tools, including genome editing toolkits, has facilitated the engineering of physiological studies of *S. thermophilus* and the generation of strains with improved technical and/or functional characteristics. Therefore, from the standpoint of the existing food industry, *S. thermophilus* genome editing will be utilized to advance our understanding of bacterial physiology. It is simple to understand that synthetic biology can considerably contribute to drug discovery, development, and other advancements given the tools available and the speed of development. Research on engineered *S. thermophilus* has been ongoing and other breakthroughs in scientific research on engineered *S. thermophilus* are expected. 

## Figures and Tables

**Table 1:** The promoters which can be used in *S. thermophilus*.

Promoters	Species of Origin	Main Features	References
STP2201	*S. thermophilus*	Active in both *S. thermophilus* and *E. coli*	[30]
P25	*S. thermophilus*	Active in both *S. thermophilus* and *E. coli*	[31]
PprtS	*S. thermophilus*	Functional in *S. thermophilus*	[33]
Native promoter library-1	*S. thermophilus*	28 promoters with different strengths, static regulation of gene expression level for metabolic engineering	[34]
PhlbA	*L. delbrueckii* subsp. *bulgaricus*	Strong and constitutive expression in *S. thermophilus*	[35]
PnisA	*L. lactis*	Inducible by nisin	[36]
Phsp16	*S. thermophilus*	Inducible by acid shock	[37]
Pshsp	*S. thermophilus*	Inducible by heat shock	[38]
Plac	*S. thermophilus*	Inducible by lactose	[39]
Promoters with ComR-box	*Streptococci* species	Inducible by ComS_17–24_	[40]

**Table 2:** The terminators of *S. thermophilus*.

Features	*S.thermophilus* CNRZ1066 Chromosome, Complete Genome	*S. thermophilus* JIM 8232, Complete Genome	*S. thermophilus* LMD-9, Complete Genome	*S. thermophilus* LMG 18,311 Chromosome, Complete Genome	*S. thermophilus* ND03 Chromosome, Complete Genome
Genes	2000	2230	1795	1973	1990
Terminators	754	807	721	738	770
GreatestΔG	604	642	569	593	621
L	598	631	566	587	595
L GreatestΔG	506	535	479	498	509
I	156	176	155	151	175
I greatestΔG	98	107	90	95	112
X	40	43	33	36	40
X greatestΔG	27	31	24	25	33
U	63	75	67	59	64
V	0	0	0	0	0
References	[42]	[42]	[42]	[42]	[42]

**Table 3:** The plasmids used for *S. thermophilus*.

Plasmid	Replicon (Selection Gene)	Size (kb)	Utilization	References
pGKV210	pND919 (*cadA, cadC*)	2.9	Heavy metal resistance, cadmium resistance	[43]
pHRM1	pSt08 (*shsp*)	6.4	Thermoresistance vector (*shsp*)	[44]
pBUL1 + pSY1	pSintA1 (*thyA*)	8.0	Thymidylate auxotrophic complementary selection marker	[45]
pNZ123	pRAF301 (*aga*)	2.5	Selection marker for α-galactosidase (aga encoding)	[46]
pER8		2.2	Cryptic plasmids of *S. thermophilus*	[47]
pER371		2.7		
pMEU5 and pMEU6	pER8 (*erm, bla*)	5.7	Shuttle vector for *E. coli* and *S. thermophilus* and other LAB	[48]
pMEU9 and pMEU10	pER8 (erm, bla, cat)	6.9		
pG + host9	pWV01 (Ts) (*erm*)	3.7	Thermosensitive vector for gene inactivation and random insertional mutagenesis	[49]
p5aGFP2201a	pMEU5a + pUCP8201 (*bla*, *erm*, *gfp*)	6.4	Shuttle vector for *E. coli*, *S. thermophilus* and other LAB	[50]
pMeu14′-1	pER371 + pUER28b (*bla*, *erm*)	5.3	Shuttle vector for *E. coli* and *S. thermophilus*	[51]
pPC418	p5aGFP2201a (*erm*, *bla*)	7.9	Pediocin expression in LAB	[52]
pPC318	p5aGFP2201a (*bla*)	9.1		
pG341a/b	pMEU5a (*bla*, *erm*)	6.4	Heterologous gene expression in *S. thermophilus* and *E. coli*	[53]
pSMQ172cat	pSMQ172 (*cat*)	5.7	Theta replication shuttle vector for E. coli, *S. thermophilus*, *S. salivarius*, and *L. lactis*	[54]
pINTRS	pWV01 (Ts) (*erm*)	5.3	Food-grade vector for chromosomic insertion of heterologous DNA or gene inactivation	[55]

**Table 4:** The endogenous plasmids of *S. thermophilus*.

Plasmid Name	Strain	Replication	Size (kb)	(G + C)%	Protein	Gene	Rep Protein	NCBI Accession	References
pSMQ172	SMQ-172	RCR	4.23	38	4	5	Rep 223 aa	NC_004958.1	[54]
pSMQ-316		θ	6.71	37.7	5	5		NC_010859.1	[54]
pCI65st	NDI-6	RCR	6.5	34.5	5	5	RepA 315 aa	AF027167.1	[57]
pND103	ST2-1		3.53	32.4	4	4		NC_004747.1	[58]
pSt0	St0	RCR	8.1	37	6	6		NC_025154	[59]
pSt04	St04	RCR	3.1					AJ242477	[60]
pSt08	St08	RCR	7.51		9	1	Rep 313 aa	AJ 239049	[60]
pSt106			5.283	36		1	Rep 287 aa	AJ 242479	[60]
pJ34	J34	RCR	3.38			1	RepA 315 aa	AJ242475	[60]
pSt22-2	St22								[60]
pER1-1		RCR	3.365		2	1	RepA 314 aa	AJ 242476	[60]
pER1-2			4.45	36.9	5	5		NC_025196.1	[60]
pt38	ST2783		2.91	32.4	5	9	Rep 311 aa	NC_005098.1	[61]
pER16			4.27		3		Rep 315 aa	AF177166	[61]
pER35	ST135		9.53	36.5	5	5	RepA 315 aa	NC_000937.1	[61]
pER36	ST136		3.5	34.4	2	2	RepA 315 aa	NC_000938.1	[62]
pK1002C2	K1002C2		3.38	35	2	2	RepA 314 aa	NC_019231.1	[63]
pK2007C6	K2007C6		2.98	35.1	2	2	RepA 314 aa	NC_019232.1	[63]
pSMQ173b	SMQ-173	RCR	4.45	37	5	5	Rep 146 aa	NC_005323.1	[64]
pSMQ-308			8.14	37.8	6	6		NC_005322.1	[64]
pSTER_A	LMD-9	RCR	4.45	37	4	4		NC_008500.1	[65]
pSTER_B	LMD-9	RCR	3.36	35.1	2	2	Rep 314 aa	NC_008501.1	[65]
pER341	ST134	RCR	2.798	33.7		2		AF019139.1	[65]
pER371	ST371		2.67	38.2	3	3	Rep 247 aa	NC_004968.1	[66]
pER13	ST113	RCR	4.14	38.4	4	4	RepB 217 aa	NC_002776.1	[67]
pSTHERMO	STH_CIRM_65		3.35	33.6	3	3	Rep	NZ_LR822016.1	[68]
pST64987	ST64987		7.98	37.5	7	7		NZ_CP049054.1	[69]
paSTHERMO	STH_CIRM_956		4.40	39.4	3	3	Rep	NZ_LR822021.1	[69]
pbSTHERMO	STH_CIRM_956		2.16	36.8	1	1	Rep	NZ_LR822022.1	[69]
pSTHERMO	STH_CIRM_998		4.40	39.3	3	3	Rep	NZ_LR822028.1	[69]
pSTHERMO	STH_CIRM_336		3.82	33.4	2	2	Rep	NZ_LR822018.1	[69]
pSTHERMO	STH_CIRM_1121		3.53	32.3	2	2	Rep	NZ_LR822038.1	[69]
pSTHERMO	STH_CIRM_67		3.35	35.5	3	3	Rep	NZ_LR824003.1	[69]
p.P3A	TK-P3A		3.50	37.3	2	3		NZ_CP045597.1	[69]
p202_03	MAG_rmk202_sterm		14.14	36.1	15	17		NZ_CP046135.1	[69]
p1	TH-4		3.36	35.1	2	3	Rep	NZ_CP102539.1	[69]
p2	TH-4		4.45	37	5	5		NZ_CP102540.1	[69]
paSTHERMO	STH_CIRM_368		4.45	39.4	3	3		NZ_LR822024.1	[70]

**Table 5:** The characteristics of *S. thermophilus* engineering strains and their application.

Bacterial Chassis	Peptide	Wild-Type/Gene Source	Expression Details	Purpose	Reference
*S. thermophilus* LMD9	L-arabinose isomerase	*Geobacillus stearothermophilus*	The plasmid pMR4	Diabetic patients	[32]
*S. thermophilus* ST128	PepA-D or papA-D (pediocin operon)	*Pediococcus acidilactici*	NICE system, under PnisA control	Against Listeria in dairy food	[36]
*S. thermophilus* ST128	Tyrosinase	*S. antibioticus*	Cloning vector pIL253	The synthesis of tyrosinase protein by genetic transformants	[82]
*S. thermophilus* SMQ-301	Galactokinase	*S. salivarius*	The plasmid pTRKL2TK, galK (galactokinase) and galM (mutarotase)	Galactose reduction in dairy products	[83]
*S. thermophilus*	Serine hydroxymethyltransferase (SHMT)	*S. thermophilus* NIZOB505	Promoter PLacA, the glyA gene	Control and improvement of acetaldehyde production in fermented (dairy) products with S. thermophilus as starter culture	[84]
Streptococcus thermophilus ST128	Bioactive peptides BL-11 and C-12	*Bos taurus*	The ST2201 promoter	Bioactive peptides from milk proteins	[85]
Streptococcus thermophilus CRL 807	Superoxide dismutase and catalase	*L. casei*	The plasmid pIL253-sodA, pIL253-mnkat	Antioxidant enzyme production to confer anti-inflammatory potential	[86]

## Data Availability

Data are available on request from the corresponding authors.
